# Pulmonary rehabilitation under supervision of health-professional at institute versus conventional exercise-based pulmonary rehabilitation at home in COPD patients: A longitudinal cohort study

**DOI:** 10.1016/j.clinsp.2024.100563

**Published:** 2025-01-28

**Authors:** Guxiang Zhang, Jiajing Yu, Zhiqiang Pei, Fei Xie, Ruiyang Ding, Lili Bao, Anyang Li

**Affiliations:** a Department of Respiratory & Critical Care Medicine, Affiliated Hospital of West Anhui Health Vocational College, Lu'an City, Anhui Province, China; b Department of Rheumatology & Immunology, Lu'an People's Hospital of Anhui Province, Lu'an City, Anhui Province, China; c Department of Imaging, Affiliated Hospital of West Anhui Health Vocational College, Lu'an City, Anhui Province, China

**Keywords:** Airflow obstruction, BODE index, Body-mass index, Chronic obstructive pulmonary disease, Dyspnea, Exercise, Health-professional, Inhaled pharmacological treatment, The Global Initiative for Chronic Obstructive Lung Disease, Pulmonary rehabilitation

## Abstract

**Objectives:**

To evaluate the efficacy and safety of pulmonary rehabilitation under the supervision of health professionals at the institute versus conventional exercise-based pulmonary rehabilitation at home in Chronic Obstructive Pulmonary Disease (COPD) patients.

**Methods:**

Patients of COPD received pulmonary rehabilitation under the supervision of a professional at the institute (PI cohort, *n* = 115) or self-driven traditional Chinese methods-based pulmonary rehabilitation at home (CE cohort, *n* = 127) or did not receive any type of pulmonary rehabilitation (ME cohort, *n* = 155). All patients received inhaled pharmacological treatment for COPD.

**Results:**

Before commencing inhaled pharmacological treatment with or without pulmonary rehabilitation (BT) COPD patients had 5 (5–4) / patient BODE (body-mass index, airflow obstruction, dyspnea, and exercise capacity) index score and 12 (13–12) / patient exacerbations (in 6-months) reported. After 6 months of inhaled pharmacological treatment for COPD with or without pulmonary rehabilitation (AT), a six-minute walking test was improved and the BODE index score and exacerbations during 6 months were decreased for patients of the PI cohort as compared to BT conditions and compared to those of the CE and ME cohorts in AT conditions (*p* < 0.05 for all). Patients of PI, CE, and ME cohorts had the risk of under treatment for <0.01 BODE index score, <1.32 BODE index score, and <3.14 BODE index score, respectively.

**Conclusions:**

Chinese patients with COPD have worse clinical conditions. After 6 months of inhaled pharmacological treatment for COPD with pulmonary rehabilitations at institutes improves the conditions of COPD patients.

## Highlights

• Guidelines recommended pulmonary rehabilitation in COPD.
• Self-driven pulmonary rehabilitation is an undertreatment for COPD patients in China.
• Chinese patients with COPD have worse clinical conditions.
• Treatment with pulmonary rehabilitation at institutes improves clinical outcomes.
• Chinese COPD patients are required to be treated as per proper guidelines.

## Introduction

Chronic Obstructive Pulmonary Disease (COPD) is a chronic lung disease that is associated with several factors.^1^ In COPD, patients have chronic airflow limitation because of airway and/or alveolar abnormalities.^2^ Pulmonary rehabilitation is an important component of the management of COPD^3^ because it is an evidence-based non-treatment intervention in symptomatic COPD patients in stable or exacerbations conditions.^4^ COPD is most prevalent in China but knowledge regarding its conditions and treatments is insufficient.^5^ In addition, in Chinese primary healthcare institutions, physicians have insufficient knowledge of pulmonary rehabilitation in COPD,^6^ Large numbers of patients with COPD are underdiagnosed and non-adherent to treatments in Chinese primary healthcare institutions.^7^ Several international guidelines recommend pulmonary rehabilitation for COPD.^8^ Chinese medicine lung rehabilitation body recommends Chinese traditional therapies (simplified Taijiquan, Baduanjin, Liuzijue, acupoint application therapy, acupuncture, Moxibustion, and pulmonary Daoyin) and the Western type modern therapies (physical exercise and relaxation therapies) to help treatment of pulmonary diseases and preserve physical and mental health.^9^ Different intensities of pulmonary rehabilitation are effective in different categories of COPD.^10^ Pulmonary rehabilitation improves the quality of life of patients with COPD.^11^ In addition, pulmonary rehabilitation improves dyspnea in routine life and decreases fat levels in the body among overweight and obese patients with compromised respiratory functions.^12^ However, the effects of pulmonary rehabilitation under the supervision of healthcare at institutes for COPD patients have not been evaluated, yet in Chinese settings. In China, there are significant discriminations among different settings for the management of COPD patients. In almost every patient in a home-based self-driven pulmonary rehabilitation is undertreatment.^7^

The objectives of retrospectively collected medical records analyses of data of COPD patients were to evaluate the short-term effects of pulmonary rehabilitation under the supervision of healthcare at institutes against self-driven conventional short-term exercise-based pulmonary rehabilitation at home against those who did not receive any type of pulmonary rehabilitation at institutes or home except follow-up visits of consultants at 45 days.

## Materials and methods

### Ethics approval and consent to participate

The designed protocols of the established study were prepared by the authors themselves and approved by the human ethics committee of the Affiliated Hospital of West Anhui Health Vocational College (Approval number 15WAHVCfA dated May 14, 2024). The study was conducted following the 2008 Declaration of Helsinki and the law of China. Informed consent was waived by the human ethics committee of the Affiliated Hospital of West Anhui Health Vocational College (because of the retrospective study).

### Inclusion criteria

Patients with reported COPD by a pulmonologist and under treatment and advised for pulmonary rehabilitation were included in the study.

### Exclusion criteria

Patients with incomplete hospital records (missing of a minimum three important parameters), patients with reported lung cancer, and patients with complicated asthma were excluded from the study.

### Treatments of COPD

Patients followed the Global Initiative for Chronic Obstructive Lung Disease (GOLD) 2019 inhaled pharmacological treatment for COPD.^13^

### Sample size calculations

The study assumed that using pulmonary rehabilitation at institutes or at home with treatments of COPD could reduce −10 % of BODE (body-mass index, airflow obstruction, dyspnea, and exercise capacity; primary outcome) index score after treatments of COPD with or without pulmonary rehabilitation of 6 months (effect size).^10^ Besides that, *α* = 0.5, *β* = 0.1, and 95 % of the Confidence Interval, using OpenEpi (Open-Source Epidemiologic Statistics for Public Health, Version. www.OpenEpi.com), a total of 105 patients required in each cohort (sample size). The differences were calculated by subtracting the mode value of the BODE index score of the ME cohort before the commencement of inhaled treatment from the BODE index score of individual patients 6 months after the commencement of inhaled pharmacological treatment for COPD with or without pulmonary rehabilitation.

### Cohorts

A total of 115 patients with COPD received pulmonary rehabilitation under the supervision of health professionals at the institutes (PI cohort). A total of 127 patients with COPD received self-driven conventional exercise-based pulmonary rehabilitation at home (CE cohort). A total of 155 patients with COPD did not receive any type of pulmonary rehabilitation at the institute or home except follow-up visits of consultants at 45 days (ME cohort). Besides pulmonary rehabilitation patients with COPD received inhaled pharmacological treatment for COPD according to GOLD 2019.^13^

### Different ways of pulmonary rehabilitation

#### Pulmonary rehabilitation at institutes

Pulmonary rehabilitation of patients with COPD at institutes performed under the supervision of healthcare professionals. Healthcare professionals followed the Chinese medicine lung rehabilitation body^9^ recommendations for pulmonary rehabilitation. Healthcare-professional was available every day for 1 h from 8 to 9am. Patients had to attend sessions of minimum 2 days in week. Healthcare professionals (nursing staff or pharmacists) were available on phone calls.

#### Pulmonary rehabilitation at home

Patients with COPD performed self-driven conventional exercise-based pulmonary rehabilitation at home. The conventional exercise included Chinese traditional therapies (simplified Taijiquan, Baduanjin, Liuzijue, acupoint application therapy, acupuncture, Moxibustion, and pulmonary Daoyin). Patients performed conventional exercises at their convinces (in the morning or evening; one hour or half an hour every day or two to three times a week).

### Outcome measures

#### Demographical and clinical characteristics

Demographical and clinical characteristics before the commencement of pulmonary rehabilitation including patients’ habits were collected from patients’ electronic records and analyzed.

#### BODE index

The primary outcome of the study was the BODE index. Body Mass Index (BMI) was calculated for patients in kg/m^2^. If BMI was >21 kg/m^2^ then the score was 0 and if BMI was 21 kg/m^2^ or less then the score was 1. The obstruction parameter of the BODE index was evaluated using the FEV1 % predicted (plethysmography was used to calculate the FEV1 % predicted).^10^ If the FEV1 % predicted was 65 % or more then the score was 0. If the FEV1 % predicted was 50–64 % then the score was 1. If the FEV1 % predicted was 36–49 % then the score was 2. For FEV1 % predicted 35 or less than the score was 3. The modified Medical Research Council (mMRC) dyspnea scale (Table 1)^10^^,^^14^ was used to evaluate the intensity of dyspnea in routine life.

**Table 1:** The modified Medical Research Council (mMRC) dyspnea scale.

Different conditions	Scale	Corresponding scale score used in BODE calculations
Breathless only with strenuous exercise	0	0
Short of breath when walking up a slight hill or hurrying on the ground	1	0
Short of breath on a large walking	2	1
Short of breath normally in walking	3	2
Short of breath during normal daily work	4	3

Conditions were self-assessed and validated by healthcare professionals.

If patients walked 350 m or more distance in 6 min then the score was 0 if they walked 250–349 m distance in 6 min then the score was 1 if they walked 250–150 m distance in 6 min then the score was 2, and for <150 m distance in 6 min, then score was 3. All three points were summed to calculate the BODE index. The score range was 0–10. Higher the score the worse the conditions.^10^

#### Six minutes walking test

The distance covered in meters in 6 min under the supervision of a healthcare professional was considered.^10^

#### Exacerbations

Severe coughing and required hospitalization were considered exacerbations.^10^

Outcome measures were evaluated after 6 months after inhaled pharmacological treatment for COPD with or without pulmonary rehabilitation at institutes.

#### Adverse effects

Any adverse effects of pulmonary rehabilitation and/or inhaled pharmacological treatment for COPD during 6 months were evaluated.

Clinical benefits for inhaled pharmacological treatment (with or without pulmonary rehabilitation)

Clinical benefits for inhaled pharmacological treatment of patients with COPD with or without pulmonary rehabilitation were evaluated as a function of the beneficial scores. The beneficial scores for inhaled pharmacological treatment of patients with COPD for different ways of pulmonary rehabilitation were calculated from the risk of under-treatment as expressed in Eq. (1). The risk of under-treatment was defined with a calculation that involves the BODE index score below which inhaled pharmacological treatment for COPD with or without pulmonary rehabilitation were not effective for patients with COPD (Eq. (2)). The BODE index score was considered as a numerical value from 0 to 10. The beneficial score for inhaled pharmacological treatment of patients with COPD for different ways of pulmonary rehabilitation is the area above the curve of the inhaled pharmacological treatment of patients with COPD with adopted pulmonary rehabilitation and the working area is the area under the curve of the inhaled pharmacological treatment of patients with COPD with adopted pulmonary rehabilitation. For all adopted pulmonary rehabilitation with inhaled pharmacological treatments, more than −10 % differences (with respect to the initial value of ME cohort patients) in the BODE index score were used as the reference standard.^15^ The differences in % in the BODE index score were calculated from Eq. (3).

(1) $$\text{Beneficial}\;\text{score}=\frac{\text{The}\;\text{number}\;\text{of}\;\text{patients}\;\text{with}\;-10\;\%\;\text{or}\;\text{more}\;\text{BODE}\;\text{index}\;\text{score}\;\text{difference}}{\text{Total}\;\text{number}\;\text{of}\;\text{patients}}+\left(\frac{\text{The}\;\text{number}\;\text{of}\;\text{patients}\;\text{with}\;\text{less}\;\text{than}\;-10\;\%\;\text{BODE}\;\text{index}\;\text{score}\;\text{difference}}{\text{Total}\;\text{number}\;\text{of}\;\text{patients}}\;\times \;\text{Risk}\;\text{of}\;\text{underttreatment}\right)$$

(2) $$\text{Risk}\;\text{of}\;\text{undertreatment}=\frac{\text{BODE}\;\text{index}\;\text{score}-10}{\text{BODE}\;\text{index}\;\text{score}}$$

(3) $$\%\;\text{difference}\;\text{in}\;\text{BODE}\;\text{index}\;\text{score}=\left(\frac{\text{BODE}\;\text{index}\;\text{score}-\text{Mode}\;\text{value}\;\text{of}\;\text{BODE}\;\text{index}\;\text{of}\;\text{ME}\;\text{cohort}\;\text{before}\;\text{treatment}}{\text{BODE}\;\text{index}\;\text{score}}\right)\times \;100$$

### Statistical analyses

InStat 3.01 was used for statistical analyses of variables. Non-normal continuous, categorial, and normal continuous variables are depicted as median with Q3–Q1 in parenthesis, frequencies with percentages in parenthesis, and mean ± Standard Deviation (SD), respectively. ꭓ^2^-test (Chi-Square test for large tables) or Fisher exact test (2 × 2 tables) was preferred for statistical analyses of categorical variables. Soup calculator® was used to calculate quartile values. Kruskal-Wallis’ test or Mann-Whitney test (between cohort) and Wilcoxon matched-pairs signed-ranks test or Friedman test (nonparametric repeated measures analysis of variance [ANOVA]) were used for statistical analysis of non-normal continuous variables. All results were considered significant if the *p*-value was <0.05.

## Results

### Study populations

**Fig. 1:**
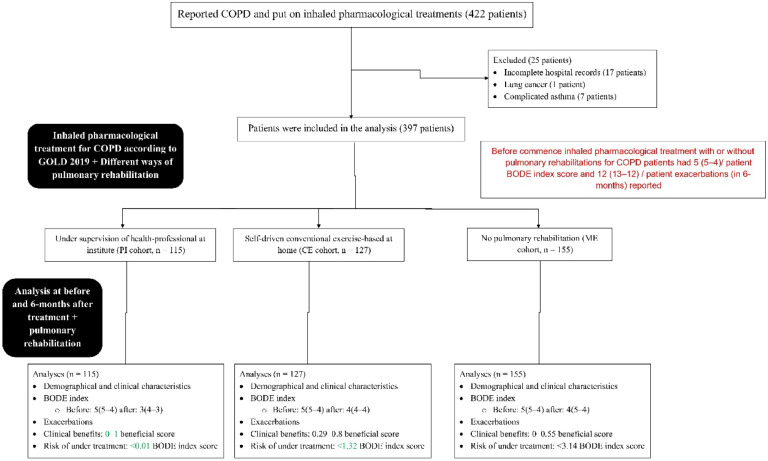
Flow diagram of retrospective analyses. The red color indicates worse parameters. The green color indicates comparatively better outcomes of treatments. COPD, Chronic obstructive pulmonary disease; GOLD, Global Initiative for Chronic Obstructive Lung Disease; BODE index, Body-mass index, airflow obstruction, dyspnea, and exercise capacity index (range 0–10, the higher the score worse the condition).

From January 15, 2020, to March 18, 2022, a total of 422 patients with reported COPD and put on inhaled pharmacological treatments at the parent hospital and the referring hospitals. Among 422 patients with COPD, 17 patients had incomplete hospital records (missing of a minimum three important parameters), one patient with reported lung cancer, and seven patients with complicated asthma. Therefore, data from these (25 patients) were excluded from the study. Variables including BODE index score (primary objective), outcome measures, adverse effects, and clinical benefits (according to primary objective) for adopted treatments were extracted from hospital records for 397 patients with COPD. The flow chart of a retrospective study is presented in Fig. 1.

### Demographical and clinical characteristics

Male to female ratio was 2/3–1/3. Patients were Han Chinese and 50 years or more years in age at the start of treatment. The distribution of patients according to GOLD categories was similar among cohorts. In addition, there were no statistical differences in the demographical and clinical characteristics before commencing pulmonary rehabilitation with inhaled pharmacological treatment for COPD among cohorts (Table 2).

**Table 2:** Demographical and clinical characteristics before commencing pulmonary rehabilitation with inhaled pharmacological treatment for COPD.

Characteristics		Total	Cohorts			Comparisons among cohort		
			PI	CE	ME			
Pulmonary rehabilitation		‒	Healthcare-professional at institutes	Self-driven at home	None			
Numbers of patients		397	115	127	155	*p*-value	Df	Test value
Gender	Male	260 (65)	75 (65)	80 (63)	105 (68)	0.7039 (ꭓ^2^ test for independence)	2	0.7022
	Female	137 (35)	40 (35)	47 (37)	50 (32)			
Age (years)		56 (60–54)	55 (60–54)	56 (60–54)	58 (61–54)	0.1037 (Kruskal-Wallis’ test)	N/A	4.532
Inhaled pharmacological treatment according to GOLD								
Group A		62 (16)	15 (13)	15 (12)	32 (21)	0.131 (ꭓ^2^ test for independence)	6	9.852
Group B		132 (33)	45 (39)	47 (37)	40 (26)			
Group C		115 (29)	30 (26)	40 (31)	45 (29)			
Group D		88 (22)	25 (22)	25 (20)	38 (24)			
Ethnicity								
Han Chinese		367 (92)	107 (93)	118 (93)	142 (92)	0.9867 (ꭓ^2^ test for independence)	6	0.3455
Mongolian		26 (7)	7 (6)	8 (6)	11 (7)			
Tibetan		4 (1)	1 (1)	1 (1)	2 (1)			
Body mass index (kg/m^2^)		25 (26–24)	25 (26–24)	25 (26–24)	25 (26–24.5)	0.1006 (Kruskal-Wallis’ test)	N/A	4.593
FEV1 % predicted		52 (54–51)	52 (54–51)	52 (54–51)	52 (55–51)	0.068 (Kruskal-Wallis’ test)	N/A	5.376
mMRC		3 (4–3)	3(4–3)	3(4–3)	3(4–3)	0.564 (Kruskal-Wallis’ test)	N/A	1.145
Six minutes walking test (m)		255 (261–249)	257 (261–245)	255 (268–248)	257 (259–249)	0.0736 (Kruskal-Wallis’ test)	N/A	5.219
BODE index score		5 (5–4)	5 (5–4)	5 (5–4)	5 (5–4)	0.2448 (Kruskal-Wallis’ test)	N/A	2.814
Exacerbations in 6 months before commencement of treatments		12 (13–12)	12 (13–12)	12 (13–12)	12 (13–12)	0.1741 (Kruskal-Wallis’ test)	N/A	3.496
Smoking	No smoker	370 (93)	112 (97)	119 (94)	140 (90)	0.0699 (ꭓ^2^ test for independence)	2	5.409
	Previous smoker	26 (7)	3 (3)	8 (6)	15 (10)			
Drinking	No drinking	381 (96)	108 (94)	121 (95)	153 (99)	0.0984 (ꭓ^2^ test for independence)	2	4.638
	Previous drinking	15 (4)	7 (6)	6 (5)	2 (1)			

Df, Degree of Freedom; COPD, Chronic Obstructive Pulmonary Disease; GOLD, Global Initiative for Chronic Obstructive Lung Disease; BODE index, Body-mass index, airflow obstruction, dyspnea (range 0–10, the higher the score worse the condition); mMRC, The modified Medical Research Council; N/A, Not Applicable.

Non-normal continuous and categorical variables are depicted as median with Q3–Q1 in parenthesis and frequencies with percentages in parenthesis.

Test value (ꭓ^2^-value for ꭓ^2^-test; Kruskal-Wallis’ statistics for Kruskal-Wallis’ test).

All results were considered significant if the *p*-value was <0.05.

Before commencing inhaled pharmacological treatment with or without pulmonary rehabilitation for COPD patients had 5 (5–4) / patient BODE index score and 12 (13–12) / patient exacerbations (in 6 months) reported. The details of the BODE index score corresponding to individual parameters before commencing pulmonary rehabilitation with inhaled pharmacological treatment for COPD are reported in Table 3.

**Table 3:** BODE index score corresponding to individual parameters before commencing pulmonary rehabilitations with inhaled pharmacological treatment for COPD.

Characteristics	Total	Cohorts		
		PI	CE	ME
Numbers of patients	397	115	127	155
Pulmonary rehabilitation	‒	Healthcare-professional at institutes	Self-driven at home	None
Body mass index (kg/m^2^)	0 (0–0)	0 (0–0)	0 (0–0)	0 (0–0)
FEV1 % predicted	1 (1–1)	1 (1–1)	1 (1–1)	1 (1–1)
mMRC	2 (3–2)	2 (3–2)	2 (3–2)	2 (3–2)
Six minutes walking test	1 (2–1)	1 (2–1)	1 (2–1)	1 (2–1)

Variables are depicted as median with Q3–Q1 in parenthesis.

BODE index, Body-mass index, airflow obstruction, dyspnea (range 0–10, the higher the score worse the condition); mMRC, The modified Medical Research Council.

### Outcome measures

#### Six minutes walking test

After 6 months of inhaled pharmacological treatment for COPD with or without pulmonary rehabilitation six-minute walking test was improved across all the cohorts (*p* < 0.05 for all, Friedman test/Dann's multiple comparisons test). A six-minute walking test was better in the PI and CE cohorts than those of the ME cohort after 6 months of inhaled pharmacological treatment for COPD (*p* < 0.05 for all, Kruskal-Wallis’ test/Dann's multiple comparisons test). In addition, the six-minute walking test was better in the PI cohort than in the CE cohort after 6 months of inhaled pharmacological treatment for COPD (*p* < 0.05 for all, Kruskal-Wallis’ test/Dann's multiple comparisons test).

#### BODE index and exacerbations

BODE index score 6 months after commencing of pulmonary rehabilitation with inhaled pharmacological treatment for COPD and exacerbations during 6 months after commencing of pulmonary rehabilitation with inhaled pharmacological treatment for COPD were fewer for patients of the PI, CE, and ME cohorts as compared to before commencing of pulmonary rehabilitation with inhaled pharmacological treatment for COPD (*p* < 0.05 for all, Friedman test/Dann's multiple comparisons test). BODE index score 6-months after commencing of pulmonary rehabilitation with inhaled pharmacological treatment for COPD and exacerbations during 6 months after commencing of pulmonary rehabilitation with inhaled pharmacological treatment for COPD were fewer for patients of the PI cohort than those of patients of the CE and ME cohorts. BODE index score and exacerbations were statistically the same for patients of the CE and ME cohorts.

The details of the BODE index score corresponding to individual parameters 6 months after the commencement of pulmonary rehabilitation with inhaled pharmacological treatment for COPD are reported in Table 4. Treatment of COPD with or without pulmonary rehabilitation of 6 months reduced the BODE index score of patients of ME, PI, and CE cohorts were −25 (0–−25), −67 (−25–−67), and −25 (−25–−25), respectively. % Differences in BODE index score after 6 months of pulmonary rehabilitation with treatment of COPD in patients of PI were higher than those of patients of ME and CE cohorts (*p* < 0.0001 for both, Kruskal-Wallis’ statistics: 112, Kruskal-Wallis’ test). However, % differences in BODE index score after 6 months of pulmonary rehabilitation with the treatment of COPD in patients of the CE cohort were statistically similar to those of the ME cohort (*p* > 0.05, Kruskal-Wallis’ statistics: 112, Kruskal-Wallis’ test).

**Table 4:** BODE index score corresponding to individual parameters 6-months after commencing of pulmonary rehabilitation with inhaled pharmacological treatment for COPD.

Characteristics	Total	Cohorts		
		PI	CE	ME
Numbers of patients	397	115	127	155
Pulmonary rehabilitation	‒	Healthcare-professional at institutes	Self-driven at home	None
Body mass index (kg/m^2^)	0 (0–0)	0 (0–0)	0 (0–0)	0 (0–0)
FEV1 % predicted	1 (1–1)	1 (1–1)	1 (1–1)	1 (1–1)
mMRC	1 (2–1)	1 (2–1)	2 (2–2)	2 (2–2)
Six minutes walking test	1 (1–1)	1 (1–1)	1 (1–1)	1 (1–1)

Variables are depicted as median with Q3–Q1 in parenthesis.

BODE index, Body-mass index, airflow obstruction, dyspnea (range 0–10, the higher the score worse the condition); mMRC, The modified Medical Research Council.

The details of outcome measures 6 months after the commencement of pulmonary rehabilitation with inhaled pharmacological treatment for COPD are reported in Table 5.

**Table 5:** Outcome measures after and during 6-months after the commencement of pulmonary rehabilitation with inhaled pharmacological treatment for COPD.

Parameters	Cohorts							Comparisons between PI and CE	
	ME	PI			CE				
Pulmonary rehabilitation	None	Healthcare-professional at institutes			Self-driven at home				
Numbers of patients	155	115	a*p*-value	Test value	127	a*p*-value	Test value	*p*-value	Test value
BODE index score	4 (5–4)	3 (4–3)	<0.001	113	4 (4–4)	>0.05	113	<0.001	113
Exacerbations	12 (13–12)	11 (12–10)	<0.001	91	12 (12–12)	>0.05	91	<0.001	91

Variables are depicted as median with Q3–Q1 in parenthesis.

a Concerning ME cohort. Kruskal-Wallis’ test was used for statistical analysis. Dann's multiple comparisons test was used for post hoc analysis. All results were considered significant if the *p*-value was <0.05. BODE index, Body-mass index, airflow obstruction, dyspnea (range 0–10, the higher the score worse the condition).

The results of the assumptions tests are presented in Table 6.

**Table 6:** Results of the assumption test.

Variables	Statical algorithm results for adopted test
Categorial variables	
For 2 × 2 tables	Fisher's exact test or Chi-Squared test (when the sample size is >40)
For large contingency tables	Chi-Squared test for independence
Demographical and clinical characteristics	
Age (years)	2 columns (*p* < 0.0001 & 0.0066) had not passed the normality test, i.e., Kruskal-Wallis’ test (nonparametric ANOVA)
Body mass index (kg/ m^2^)	3 columns (*p* = 0.0406; 0.0142; 0.0105) had not passed the normality test, i.e., Kruskal-Wallis’ test
FEV1 % predicted (absolute value)	3 columns (*p* = 0.0012; 0.0083; 0.0062) had not passed the normality test, i.e., Kruskal-Wallis’ test
mMRC (absolute value) score	3 columns (*p* < 0.0001 for all) had not passed the normality test, i.e., Kruskal-Wallis’ test
Six minutes walking test (m)	3 columns (*p* = 0.0021;<0.0001;<0.0001) had not passed the normality test, i.e., Kruskal-Wallis’ test
Outcome measures	
BODE index score before and 6-months after the commencement of pulmonary rehabilitation with inhaled pharmacological treatment for COPD within and between cohorts	3 columns (*p* < 0.0001 for all) had not passed the normality test, i.e., Kruskal-Wallis’ test between cohorts and Friedman test within
Exacerbations in 6-months before the commencement of treatments within and between cohorts	3 columns (*p* < 0.0001 for all) had not passed the normality test, i.e., Kruskal-Wallis’ test between cohorts and Friedman test within
Six minutes walking test (m) 6-months after the commencement of pulmonary rehabilitation with inhaled pharmacological treatment for COPD within and between cohorts	3 columns (*p* < 0.0001 for all) had not passed the normality test, i.e., Kruskal-Wallis’ test between cohorts and Friedman test within
Exacerbations during 6-months after the commencing of pulmonary rehabilitation with inhaled pharmacological treatment for COPD within and between cohorts	3 columns (*p* < 0.0001 for all) had not passed the normality test, i.e., Kruskal-Wallis’ test between cohorts and Friedman test within

COPD, Chronic obstructive pulmonary disease; mMRC, The modified Medical Research Council; BODE index, Body-mass index, airflow obstruction, dyspnea, and exercise capacity index; ANOVA, Analysis of Variance.

Clinical benefits for inhaled pharmacological treatment (with or without pulmonary rehabilitation)

**Fig. 2:**
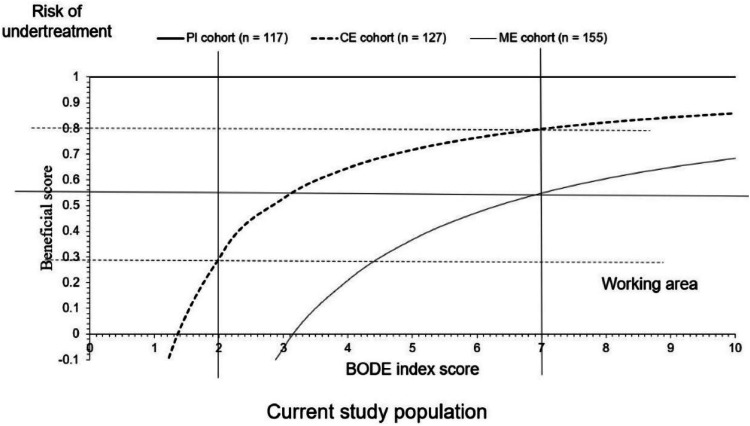
The graphical presentation of clinical benefits of inhaled pharmacological treatment (with or without pulmonary rehabilitation) for patients of different cohorts. BODE index, Body-mass index, airflow obstruction, dyspnea, and exercise capacity index (range 0–10, the higher the score worse the condition).

Clinical benefits for patients of PI, CE, and ME cohorts had 0–1, 0.29–0.8, and 0–0.55 beneficial scores for inhaled pharmacological treatment (with or without pulmonary rehabilitation), respectively. Patients of PI, CE, and ME cohorts had a risk of under treatment for <0.01 BODE index score, <1.32 BODE index score, and <3.14 BODE index score, respectively. Working areas for the current study population were 2–7 BODE index score, 2–7 BODE index score, and 3.14–7 BODE index score, respectively for Patients of PI, CE, and ME cohorts. The details of the clinical benefits of inhaled pharmacological treatment (with or without pulmonary rehabilitation) are presented in Table 7. The graphical presentation of the clinical benefits of inhaled pharmacological treatment (with or without pulmonary rehabilitation) for patients of different cohorts is presented in Fig. 2.

**Table 7:** Clinical benefits for inhaled pharmacological treatment (with or without pulmonary rehabilitation).

Parameters	Cohorts		
	PI cohort	CE cohort	ME cohort
Pulmonary rehabilitation	Healthcare-professional at institutes	Self-driven at home	None
Total number of patients	115	127	155
Patients with ≥ −10 % BODE index score differences	115	109	106
Patients with<−10 % BODE index score differences	0	18	49
BODE index score	Beneficial scores for patients		
0.01	1	−140.73	−315.13
1	1	−0.42	−2.16
2	1	0.29	−0.58
3	1	0.53	−0.05
4	1	0.65	0.21
5	1	0.72	0.37
6	1	0.76	0.47
7	1	0.8	0.55
8	1	0.82	0.60
9	1	0.84	0.65
10	1	0.86	0.68
Clinical benefits (beneficial score)	0–1	0.29–0.8	0–0.55
Working area for the current study population	2–7 BODE index score	2–7 BODE index score	3.14–7 BODE index score
Risk of under-treatment	< 0.01 BODE index score	< 1.32 BODE index score	< 3.14 BODE index score

BODE index, Body-mass index, airflow obstruction, dyspnea, and exercise capacity index (range 0–10, the higher the score worse the condition).

#### Adverse effects

Patients mainly suffered from headaches, nausea, skin rash, and shortness of breath during six months of inhaled pharmacological treatment for COPD with or without pulmonary rehabilitation. Adverse effects during 6 months of inhaled pharmacological treatment for COPD with or without pulmonary rehabilitations were comparable across all cohorts (*p* > 0.05 for all, Table 8). Beyond these adverse events, a few events were not noticed, for example, high heart rate, and body aches (because patients did not notice such adverse effects strongly; adverse effects were subjective).

**Table 8:** Adverse effects during 6 months of inhaled pharmacological treatment for COPD with or without pulmonary rehabilitation.

Event	Cohorts									Comparisons between PI and CE		
	ME	PI				CE						
Pulmonary rehabilitation	None	Healthcare-professional at institutes				Self-driven at home						
Numbers of patients	155	115	a*p*-value	Test value	95 % CI	127	a*p*-value	Test value	95 % CI	*p*-value	Test value	95 % CI
Headache	5 (3)	7 (5)	0.3713	0.7167	0.364–1.411	8 (6)	0.2612	0.6897	0.3439–1.383	0.9999	0.9809	0.5613–1.714
Dizziness	3 (2)	5 (4)	0.2917	0.6464	0.2626–1.591	5 (4)	0.4741	0.676	0.2745–1.664	0.9999	1.055	0.5591–1.989
Sore throat	9 (6)	7 (6)	0.9999	0.9786	0.6271–1.527	7 (6)	0.9999	1.025	0.6562–1.600	0.9999	1.056	0.6142–1.814
Nausea	10 (6)	8 (7)	0.9999	0.9655	0.6302–1.479	8 (6)	0.9999	1.011	0.6596–1.551	0.9999	1.056	0.6348–1.757
Upper respiratory infections	4 (3)	2 (2)	0.9999	1.166	0.6556–2.072	1 (1)	0.383	1.468	0.9345–2.305	0.6056	1.41	0.6263–3.174
Urinary tract infection	2 (1)	1 (1)	0.9999	1.163	0.5191–2.607	1 (1)	0.9999	1.216	0.5422–2.726	0.9999	1.053	0.2615–4.237
Cramps	25 (16)	20 (17)	0.7423	0.9465	0.7132–1.256	22 (17)	0.7492	0.9456	0.7072–1.264	0.9999	1.003	0.7071–1.421
Skin rash	12 (8)	10 (9)	0.8242	0.946	0.6365–1.406	8 (6)	0.8163	1.099	0.7558–1.599	0.6249	1.185	0.7662–1.833
Shortness of breath	8 (5)	6 (5)	0.9999	0.9951	0.6245–1.586	7 (6)	0.9999	0.9687	0.5960–1.575	0.9999	0.9697	0.5307–1.772

Patients had one or more adverse events during the study period.

Variables are depicted as frequencies with percentages in parentheses.

a Concerning ME cohort. Fischer's exact test was used for statistical analysis. All results were considered significant if the *p*-value was <0.05. CI, Confidence interval (using the approximation of Katz.). Test value (relative risk for Fisher's exact test).

## Discussions

Before commencing inhaled pharmacological treatment with or without pulmonary rehabilitation for COPD patients had 5 (5–4) / patient BODE index score and 12 (13–12)/ patient exacerbations (in 6 months) reported. In China, general practitioners are unable to understand COPD,^16^ and do not prescribe treatment as per GOLD guidelines.^17^ The lack of adequate knowledge and guidelines for treatments for COPD in China makes conditions worse for COPD patients. Chinese patients with COPD have worse clinical conditions.

The six-minute walking test, BODE index score, exacerbations during 6 months, clinical benefits for patients, and risk of undertreatment were better in the PI cohort than those of the CE and ME cohorts after 6 months of inhaled treatment. The results of outcome measures of the current study are consistent with those of randomized trials.^3^^,^^10^^,^^12^ Pulmonary rehabilitation at institutes improves lung capacity and six-minute walking test.^3^ Pulmonary rehabilitation at institutes improves the strength and force of the respiratory muscles.^18^ This leads to improved other outcome measures after 6 months of inhaled treatment with pulmonary rehabilitation. After 6 months of inhaled pharmacological treatment for COPD with pulmonary rehabilitations at institutes improves the clinical conditions of patients.

After 6 months of inhaled pharmacological treatment for COPD with or without pulmonary rehabilitation, a 6-minute walking test was improved and the BODE index score and exacerbations during 6 months were decreased across all the cohort. These results are consistent with those of a trial.^3^ This effect was due to inhaled pharmacological treatment as per GOLD 2019 guidelines. Chinese COPD patients are required to be treated as per proper guidelines.

The details of the comparative studies on pulmonary rehabilitation in COPD patients in different settings are presented in Table 9.

**Table 9:** Comparative studies on pulmonary rehabilitation in COPD patients in different settings.

Study	Published year	Study population	Sample size (n; patients)	Age (years)	Follow-up
Randomized controlled trial, Cameron-Tucker, et al.^3^	2014	Australian	84	65.8 ± 9.35	6-weeks
Multicenter prospective observational study, Zeng, et al.^7^	2020	Chinese	4796	64.5 ± 8.9	1-year
Single-blinded, randomized, controlled trial He, et al.^10^	2019	Chinese	217	65.3 ± 6.2	20 weeks
Prospective study, Huivaniuk et al.^12^	2022	Ukraine	40	64.86 ± 9.81	6-months
Observational study, Kerti et al.^18^	2018	Hungarian	327	64 ± 8	4-weeks
Multicenter study, Sandoz et al.^19^	2017	Austria	141	> 18	12-year

The study shows novel advantages of pulmonary rehabilitation in COPD patients. The control group consists of the patients treated with traditional Chinese methods. Part of the conclusion is related to the results of the study. However, there are so many limitations of the study, for example, the conclusion is strictly related to Chinese populations. Retrospective medical records analyses only and lack of trial. Anxiety and depression are important parameters of patients with COPD who received treatment in follow-up.^19^ However, the current study has not evaluated the psychological aspects of patients.

## Conclusions

Chinese patients with COPD have worse clinical conditions. After 6 months of inhaled pharmacological treatment for COPD with pulmonary rehabilitations at institutes improves the clinical conditions of patients. Chinese COPD patients are required to be treated as per proper guidelines.

## List of submission

COPD, Chronic obstructive pulmonary disease; GOLD, Global Initiative for Chronic Obstructive Lung Disease; BODE index, Body-mass index, airflow obstruction, dyspnea, and exercise capacity index; mMRC, The modified Medical Research Council, SD, Standard deviation; ꭓ^2^-test, Chi-Square test; PI cohort, Patients of COPD received pulmonary rehabilitation under supervision of health-professional at institute + inhaled pharmacological treatment for COPD according to GOLD 2019; CE cohort, Patients of COPD received self-driven conventional exercise-based pulmonary rehabilitation at home + inhaled pharmacological treatment for COPD according to GOLD 2019; ME cohort, Patients of COPD did not receive any type of pulmonary rehabilitation at institute or at home except follow-up visits of consultants at 45 days + inhaled pharmacological treatment for COPD according to GOLD 2019; ANOVA, Analysis of variance; BT, Before commence inhaled pharmacological treatment with or without pulmonary rehabilitations; AT, After 6 months of inhaled pharmacological treatment for COPD with or without pulmonary rehabilitation.

## Availability of data and materials

The datasets used and analyzed during the current study are available from the corresponding author upon reasonable request.

## Authors’ contributions

All the authors have read and approved the manuscript for publication. GZ was the project administrator, and contributed to the supervision, resources, validation, and literature review of the study. JY contributed to the software, methodology, conceptualization, supervision, and literature review of the study. ZP contributed to the investigation, methodology, literature review, supervision, and software of the study. FX contributed to the methodology, resources, validation, supervision, and literature review of the study. RD contributed to the methodology, formal analysis, data curation, supervision, and literature review of the study. LB contributed to the methodology, literature review, resources, software, and data curation of the study. AL contributed to resources, methodology, formal analysis, methodology, software, visualization, and literature review of the study and drafted and edited the manuscript for intellectual content. All authors agree to be accountable for all aspects of the work, ensuring its integrity and accuracy.

## Conflicts of interest

The authors declare that they have no conflicts of interest or any other competing interests regarding the results and/or discussion reported in the research.
